# MADS-domain transcription factor AGAMOUS LIKE-9 participates in the gibberellin pathway to promote bud dormancy release of tree peony

**DOI:** 10.1093/hr/uhaf043

**Published:** 2025-02-10

**Authors:** Niu Demei, Liu Fang, Gao Linqiang, Zhang Huailong, Liu Naibin, Zhang Lu, Yuan Yanchao, Liu Chunying, Gai Shupeng, Zhang Yuxi

**Affiliations:** College of Life Sciences, Qingdao Agricultural University, Qingdao 266109, Changcheng Road 700, China; University Key Laboratory of Plant Biotechnology in Shandong Province, Qingdao 266109, Changcheng Road 700, China; College of Life Sciences, Qingdao Agricultural University, Qingdao 266109, Changcheng Road 700, China; University Key Laboratory of Plant Biotechnology in Shandong Province, Qingdao 266109, Changcheng Road 700, China; College of Life Sciences, Qingdao Agricultural University, Qingdao 266109, Changcheng Road 700, China; University Key Laboratory of Plant Biotechnology in Shandong Province, Qingdao 266109, Changcheng Road 700, China; College of Life Sciences, Qingdao Agricultural University, Qingdao 266109, Changcheng Road 700, China; University Key Laboratory of Plant Biotechnology in Shandong Province, Qingdao 266109, Changcheng Road 700, China; College of Life Sciences, Qingdao Agricultural University, Qingdao 266109, Changcheng Road 700, China; University Key Laboratory of Plant Biotechnology in Shandong Province, Qingdao 266109, Changcheng Road 700, China; College of Life Sciences, Qingdao Agricultural University, Qingdao 266109, Changcheng Road 700, China; University Key Laboratory of Plant Biotechnology in Shandong Province, Qingdao 266109, Changcheng Road 700, China; College of Life Sciences, Qingdao Agricultural University, Qingdao 266109, Changcheng Road 700, China; University Key Laboratory of Plant Biotechnology in Shandong Province, Qingdao 266109, Changcheng Road 700, China; College of Life Sciences, Qingdao Agricultural University, Qingdao 266109, Changcheng Road 700, China; University Key Laboratory of Plant Biotechnology in Shandong Province, Qingdao 266109, Changcheng Road 700, China; College of Life Sciences, Qingdao Agricultural University, Qingdao 266109, Changcheng Road 700, China; University Key Laboratory of Plant Biotechnology in Shandong Province, Qingdao 266109, Changcheng Road 700, China; College of Life Sciences, Qingdao Agricultural University, Qingdao 266109, Changcheng Road 700, China; University Key Laboratory of Plant Biotechnology in Shandong Province, Qingdao 266109, Changcheng Road 700, China

## Abstract

Bud dormancy, which serves as a survival mechanism during winter, is crucial for determining the timing and quality of flowering in many perennial woody plants, including tree peony. The gibberellin (GA) signaling pathway participates in breaking bud dormancy in tree peony. Specifically, PsRGL1, a key DELLA protein, is a negative regulator in this process. MADS-box family members participate in plant growth and development regulation. In this study, a MADS-domain transcription factor, AGAMOUS-LIKE 9 (PsAGL9), was identified as a candidate interaction protein of PsRGL1 using a pull-down assay coupled with liquid chromatography–tandem mass spectrometry. *PsAGL9* expression was induced by chilling and exogenous GA_3_. Yeast two-hybrid (Y2H), pull-down, and luciferase complementation assays (LCAs) confirmed that PsAGL9 interacted with PsRGL1. *PsAGL9* overexpression significantly promoted dormancy break and upregulated the expression of marker genes such as *PsBG6*, *PsBG9*, *PsEBB1*, *PsEBB3*, and *PsCYCD*, suggesting a potential regulatory function of PsAGL9. Classical and nonclassical CArG motifs were identified in the promoter regions of *PsCYCD* and *PsEBB3,* respectively. Yeast one-hybrid (Y1H), electrophoretic mobility shift (EMSA), and dual-luciferase assays confirmed that PsAGL9 directly bound to and activated *PsCYCD* and *PsEBB3* expression, and PsRGL1 abolished the DNA-binding activity of PsAGL9. Furthermore, interaction proteins of PsAGL9 were screened, and MADS-box members PsAGL9, PsAGL6, and PsPI were identified. Y2H, LCA, and pull-down assays confirmed that PsAGL9 formed both homodimers and heterodimers, and heterodimers further promoted target gene expression. This study provides an in-depth exploration of the GA pathway and elucidates a novel pathway, PsRGL1-PsAGL9-*PsCYCD*, involved in regulating dormancy break in tree peony.

## Introduction

Perennial woody plants in temperate zones transition from growth to dormancy in response to seasonal changes, including low temperature and short photoperiod. Bud dormancy is generally defined as a period when meristem activity is suspended and buds cannot respond to growth-promoting signals until growth resumes [1, 2]. Bud endodormancy is a type of growth arrest regulated by the dormant organ itself, and under this state, even the appropriate conditions cannot restore growth [3]. This is also a survival mechanism in winter. Endodormancy is divided into three stages: induction, maintenance, and release [3], and chilling accumulation is the primary factor influencing the release of endodormancy [4–6]. Currently, global warming during winter leads to insufficient chilling exposure, causing incomplete dormancy release, which markedly reduces the bud burst rate, flowering quality, and yield [7]. Therefore, understanding the regulatory mechanism of bud endodormancy is essential.

Plant hormones also influence bud endodormancy release. Gibberellin (GA) synthesis and the GA signaling pathway are known to participate in dormancy release. For example, exogenous GAs significantly promote bud dormancy release [8–10]. In pear, *GAST1*, a GA-stimulated transcript, is rapidly upregulated during dormancy release, accompanied by increase in *GA20ox* expression and active GA levels [11]. DELLA proteins act as central repressors of GA-mediated regulation of dormancy. In peach, DELLA2 participates in the regulation of bud endodormancy release [12]. *RGA-LIKE2* (*RGL2*) inhibits bud dormancy in Japanese apricot [13]. In tree peony, PsRGL1 negatively regulates bud dormancy release [14]. These findings highlight the role of GA signaling during dormancy release in perennial woody plants, although the mechanisms remain incompletely understood. Due to the lack of a DNA-binding domain in DELLA proteins, transcription factors interacting with them, such as MADS-domain transcription factors, are increasingly gaining attention.

The MADS-box family, widely distributed in the plant kingdom, is divided into two types based on structural components. Among them, Type II proteins contain MADS (M), intervening (I), and keratin-like (K) domains in the central region, and a C domain. The MADS-box family plays important roles in many developmental processes. In particular, its role in the ABCDE model of flower development is well known [15–18]. MADS-box family members participate in dormancy transition. For instance, dormancy-associated MADS-box (DAM), initially identified in the evergrowing peach mutant, is downregulated during dormancy release with prolonged chilling exposure [4, 19]. In *Chimonanthus praecox*, *fruitfull* (*FUL*), sepallatas (*SEPs*), and agamous-like 6 (*AGL6*) regulate dormancy release [20]. Short vegetative phase (*SVP*) regulates bud dormancy by interacting with SEP and AP1 in sweet cherries [21]. Recently, a member of the MADS-box family, suppressor of constans overexpression 1 (PsSOC1), has been found to interact with DELLA protein (PsRGL1) to accelerate bud dormancy release in tree peony [22]. Collectively, these findings highlight the important roles of MADS-box genes during the dormancy process.

The transition from dormancy to active growth is characterized by the resumption of cell proliferation [23]. The cell cycle of dormant buds is generally arrested in the G1 phase [24, 25], and D-type cyclins (CYCDs) play a key role in the transition from the G1 to S phase. In poplar, early bud-break3 (EBB3) directly activates the expression of *CYCD3.1* [24] and accelerates bud dormancy release by resuming the cell cycle in the apical meristem tissue [26]. Many CYCD genes, including *CYCD1–3*, are upregulated in *EBB1*-overexpressed (OE) poplar [27]. In GA-induced bud endodormancy release in tree peony, *PsCYCD* (*PsCYCD3.3*) responds to exogenous GAs and promotes bud dormancy release by rebooting cell proliferation [9].

Tree peony (*Paeonia suffruticosa* Andr.) is very popular because of its high ornamental value [28], and its bud dormancy type belongs to the endodormancy category, which is similar to that of other perennial plants. However, insufficient chilling accumulation in warm winters reduces the flowering quality and seeding rate, severely impacting the plant’s economic value. Therefore, understanding the mechanism underlying endodormancy release is crucial for the sustainable development of the tree peony industry. Over the past 20 years, we have studied morphological changes during dormancy release [28] and identified many differentially expressed genes, proteins, microRNA, and metabolites [29–33]. We propose that activation of the GA pathway is the key to releasing bud endodormancy [30], and we have confirmed that PsRGL1 is a negative regulator of endodormancy release [14]. However, the molecular mechanism of the GA signaling pathway in regulating the release of tree peony bud dormancy remains poorly understood.

In this study, the interacting proteins of PsRGL1 were identified, revealing the E-class MADS-domain transcription factor, PsAGL9. Overexpression of *PsAGL9* facilitated dormancy release and increased the relative expression levels of marker genes related to dormancy release, including *PsBG6*, *PsBG9*, *PsEBB1*, *PsEBB3*, and *PsCYCD*. PsAGL9 directly bound to and activated the expression of *PsCYCD* and *PsEBB3*, while PsRGL1 abolished the DNA-binding activity of PsAGL9*.* PsAGL6 and PsPI formed heterodimers with PsAGL9, and these heterodimers further promoted expression of the target genes. This study revealed a novel component of the GA pathway for breaking bud dormancy in tree peony.

## Results

### PsAGL9 is an interaction protein of PsRGL1

**Figure 1:**
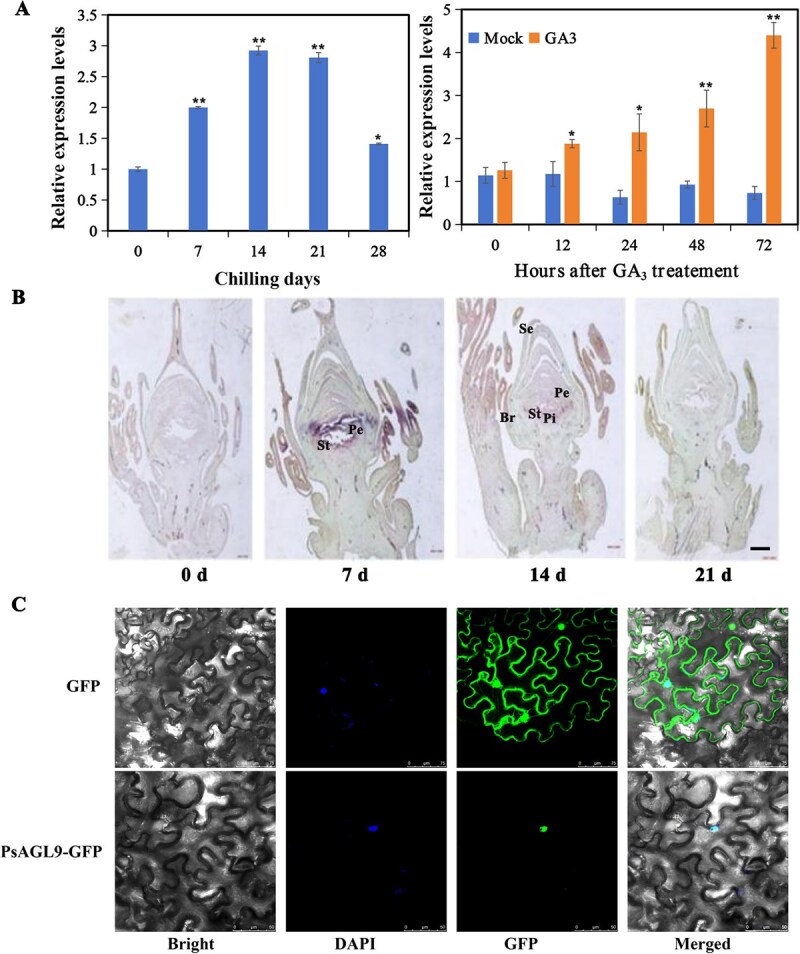
Expression of *PsAGL9* and subcellular localization. A. *PsAGL9* expression during chilling- and exogenous GA-induced bud dormancy release. Error bars for all data represented the error value from three replicates, and asterisks represented statistically significant differences (^*^P < 0.05, ^**^P < 0.01). B. *In situ* hybridization analysis of *PsAGL9* in longitudinal sections of buds chilled for 0, 7, 14, and 21 days (scale bar = 2 mm). St, stamen; Pe, petal; Br, bract; Pi, pistil; Se, sepal. C. Subcellular localization of PsAGL9 observed using fluorescence microscopy at an excitation wavelength of 488 nm. GFP, green fluorescence protein; DAPI, 4′,6-diamidino-2-phenylindole.

In a recent study, we identified, PsRGL1, a pivotal DELLA protein, as a negative regulator of bud dormancy release in tree peony [14]. Because DELLA proteins lack DNA-binding domains, DELLA-binding transcription factors have attracted considerable attention. To further screen for proteins that interact with PsRGL1, the pGEX-4 T-1 (GST)-PsRGL1 fusion protein was purified and coincubated with the total proteins from mixed buds after different chilling treatments. Following pull-down assays coupled with liquid chromatography–tandem mass spectrometry (LC–MS/MS) analysis, several potential interacting proteins were identified, including two MADS-box proteins. A BLAST search against the tree peony genome and GenBank databases showed that these proteins clustered into the SOC1 and AGL9 branches [34]. Among them, *SOC1* has been shown to accelerate endodormancy release in tree peony [22]. The open reading frame (ORF) of another MADS-box gene was 731 bp and it encoded 243 amino acids with MADS- and K-box domains (GenBank accession number: AEE60891.1; Fig. S1). Phylogenetic analysis showed that it first clustered into a branch with PpAGL9 (85.77% identity) and MdAGL9 (81.40% identity) (Figs S1 and S2). Therefore, it was named PsAGL9. Reverse transcription quantitative polymerase chain reaction (qRT-PCR) revealed that *PsAGL9* responded to early chilling, was persistently upregulated with prolonged chilling exposure, and was also induced by exogenous GA_3_ (Fig. 1A), which suggested that it might be involved in the GA pathway to regulate the breaking of bud dormancy in tree peony. The spatiotemporal expression pattern of *PsAGL9* was determined using *in situ* hybridization. High levels of *PsAGL9* transcripts were found in the stamen and petals after 7 and 14 days of chilling, followed by a decrease after 21 days (Fig. 1B). In addition, the expression level of *PsAGL9* was highest in the stamens and petals, and lowest in the roots at the early stage of flowering (Fig. S3). Notably, higher expression levels of *PsAGL9* were observed in chilled buds (Fig. S3), which was consistent with the *in situ* hybridization results. These findings suggested that *PsAGL9* might be involved in the regulation of flower organ development and bud dormancy.

Subcellular localization prediction of PsAGL9 indicated its potential nuclear localization. To confirm this prediction, the *35S::PsAGL9*-GFP vector was used to transform *Nicotiana benthamiana* leaves. In contrast to the control group, green fluorescence protein (GFP) fluorescence was restricted to the nucleus in the treated group, and colocalized with 4′,6-diamidino-2-phenylindole (DAPI)-stained nuclear DNA (Fig. 1C).

**Figure 2:**
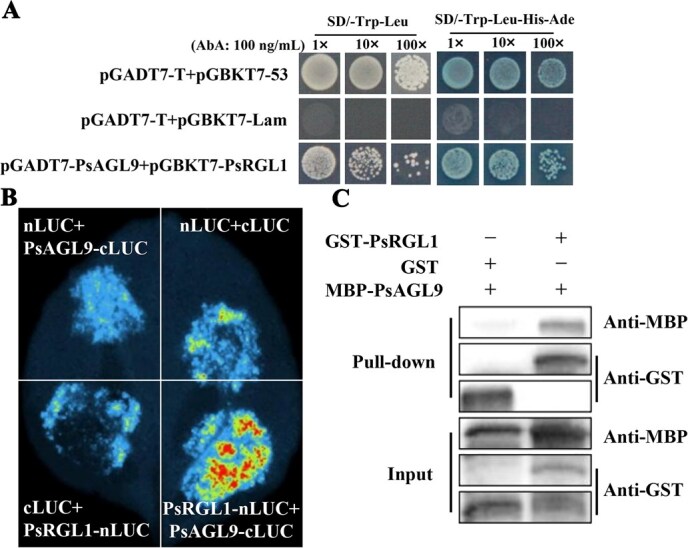
Identification of the interaction between PsAGL9 and PsRGL1. A. Y2H assay showing the interaction between PsAGL9 and PsRGL1. A fragment of PsRGL1 lacking the DELLA domain was inserted into the pGBKT7 vector, and the *PsAGL9* ORF was cloned into the pGADT7 vector. pGADT7-PsAGL9 + pGBKT7-PsRGL1 cotransformants were cultured on SD/−Trp-Leu and SD/−Trp-Leu-His-Ade/X-a-Gal supplemented with 100 ng/ml AbA. The combination of pGADT7-T + pGBKT7-53 was used as a positive control, and pGADT7-T + pGBKT7-Lam was used as a negative control. B. LCAs demonstrated the interaction between PsAGL9 and PsRGL1. The ORF of *PsAGL9* was cloned into the cLUC vector, and the *PsRGL1* ORF was ligated to the nLUC vector. Combinations containing empty nLUC or empty cLUC were used as control groups, and nLUC + cLUC was used as a negative control. C. The interaction between PsAGL9 and PsRGL1 was confirmed using *in vitro* pull-down assays. PsAGL9 fused with an MBP tag was incubated with GST-PsRGL1 or GST (control). The signal was detected using immunoblotting with anti-MBP or anti-GST antibodies.

Yeast two-hybrid system (Y2H) assays were performed to assess the interactions between PsAGL9 and PsRGL1. Considering that PsRGL1 exhibited robust self-transactivation activity at its N-terminus, a fragment of PsRGL1 lacking the DELLA domain was inserted into the pGBKT7 vector. Y2H results indicated that cotransformed yeast cells containing pGADT7-PsAGL9 and pGBKT7-PsRGL1 could grow in SD/−Trp-Leu-His-Ade medium, indicating an interaction between PsAGL9 and PsRGL1 (Fig. 2A). To further verify this interaction, a combination of PsRGL1-nLUC and PsAGL9-cLUC was coinfiltrated into *N. benthamiana* leaves. The PsRGL1-nLUC + PsAGL9-cLUC combination exhibited stronger fluorescence than the controls (Fig. 2B). In addition, GST-PsRGL1 and pMAL-c5x (MBP)-PsAGL9 fusion proteins were purified. When GST-PsRGL1 and MBP-PsAGL9 fusion proteins were coincubated, a strong signal was observed in the pull-down assay (Fig. 2C). These findings confirmed that PsAGL9 interacted with PsRGL1 as an important component in the GA signaling pathway.

### *PsAGL9* promotes bud dormancy release

**Figure 3:**
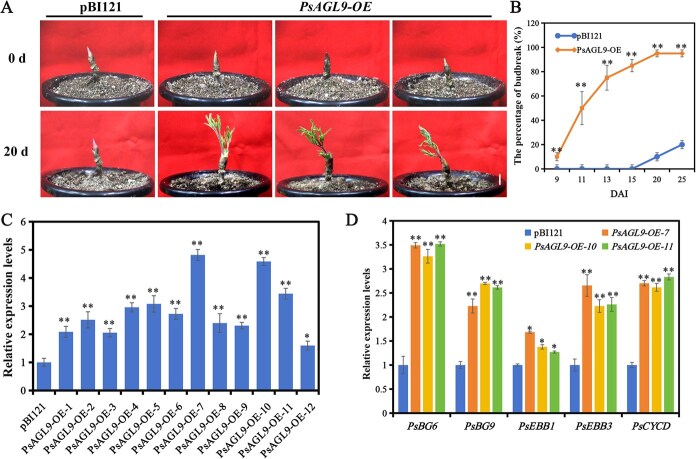
Overexpression of *PsAGL9*-promoted bud dormancy release in tree peony. A. Morphological changes in *PsAGL9*-OE buds 20 DAI. Scale bar, 1.0 cm. B. The budbreak percentage of *PsAGL9*-OE buds and control buds. C. The relative expression levels of *PsAGL9* in *PsAGL9*-OE buds after 7 DAI determined using qRT-PCR. Buds transformed with the empty pBI121 vector were used as controls. D. Relative expression levels of marker genes associated with tree peony dormancy release, including *PsEBB1*, *PsEBB3*, *PsBG6*, *PsBG9*, and *PsCYCD*, in *PsAGL9*-OE buds after 7 DAI, determined using qRT-PCR. Error bars for all data represented the error value from three replicates, and asterisks represented statistically significant differences (^*^P < 0.05, ^**^P < 0.01).

To confirm the function of *PsAGL9*, the construct pBI121-*PsAGL9* was used to infect 10-day-chilled buds. Seven days after infection (DAI), 12 buds were used to assess the transformation efficiency using qRT-PCR, and *PsAGL9* expression levels were found to be significantly higher in pBI121-*PsAGL9-*transformed buds than those in control buds (buds transformed with the empty pBI121 vector) (Fig. 3C). In *PsAGL9*-OE-7, 10, and 11 buds, the expression levels of dormancy-release-related genes, such as *PsCYCD*, *PsEBB1*, *PsEBB3*, *PsBG6*, and *PsBG9*, were markedly upregulated (Fig. 3D). At 9 DAI, *PsAGL9*-OE buds exhibited earlier budbreak than the control buds. The budbreak rate reached 85% at 15 DAI, whereas control buds began to show budbreak after 20 DAI (Fig. 3A). These results indicated that *PsAGL9* facilitated bud dormancy release.

### PsAGL9 directly binds to and activates the expression of *PsCYCD* and *PsEBB3*

*PsAGL9* overexpression led to the upregulation of marker genes, suggesting that PsAGL9 might regulate their expression. It is well known that MADS proteins bind to ‘CArG-boxes’. Therefore, the promoter sequences of these five genes were cloned and analyzed, and a classical CArG motif (CCAATTTTGG) was found exclusively in the *PsCYCD* promoter from −1308 to −1317*.* qRT-PCR results showed that *PsCYCD* was significantly upregulated in tree peony after chilling and GA_3_ treatment (Fig. S4).

**Figure 4:**
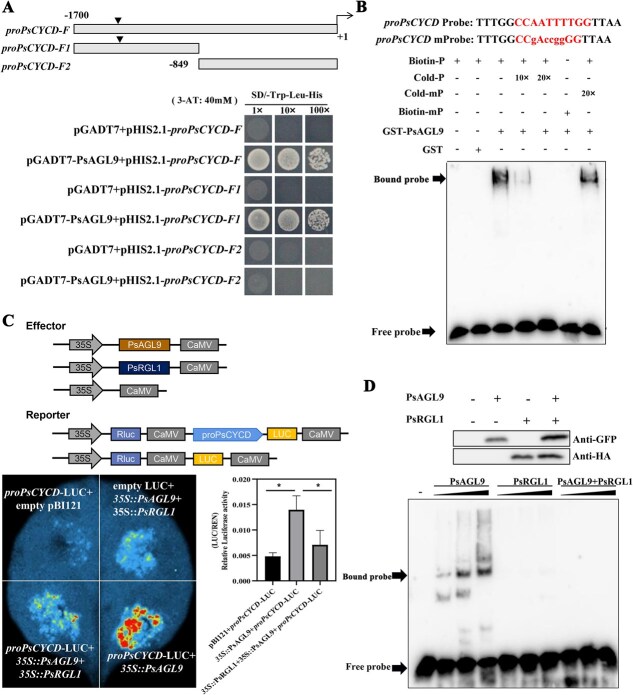
PsAGL9 directly activated the expression of *PsCYCD* by binding to its promoter*.* A. PsAGL9 bound to the fragment of the *PsCYCD* promoter containing a CArG motif, as shown in Y1H assays on SD/−Trp-Leu-His medium supplemented with 40 mM 3-AT. Diagram of *PsCYCD* promoter truncations; *proPsCYCD*-F**, the full-length *PsCYCD* promoter; *proPsCYCD-F1*, a fragment of the *PsCYCD* promoter containing a CArG motif; and *proPsCYCD-F2*, a fragment of the *PsCYCD* promoter without CArG motif. The triangle marked the site of the CArG motif. B. EMSAs showed PsAGL9 binding to the *PsCYCD* promoter fragment with a CArG motif. The lowercase letters represented the mutant bases in the CArG motif. -, incubation without protein extract. 10× and 20× represented the addition of 10- and 20-fold concentrations, respectively, of the cold probe (cold-P) or mutant cold probe (cold-mP). C. The effect of the interaction between PsAGL9 and PsRGL1 on the expression of *PsCYCD* using dual-luciferase assays*. Agrobacteria* carrying the indicated combinations were injected into tobacco leaves, and luciferase (LUC) images were captured for 72 h after injection. The empty pBI121 construct was used as an internal control. Relative luciferase activity was measured using the ratio of LUC to renilla (REN). Each value represented the mean of three biological replicates, and the vertical bars represented the mean ± standard error (SE). Asterisks represented statistically significant differences (*P < 0.05). D. EMSAs confirmed that the interaction of PsAGL9 and PsRGL1 inhibited the DNA-binding ability of PsAGL9. A DNA probe with a CArG motif in the *PsCYCD* promoter was incubated with increasing amounts of purified proteins as indicated. The abundance of PsAGL9 and PsRGL1 proteins in these extracts was evaluated by using western blotting with anti-GFP and anti-HA antibodies. Total proteins were extracted from *N. benthamiana* leaves infiltrated with *Agrobacterium* strains containing the PsAGL9-GFP and HA-PsRGL1 constructs, or a 1:1 mixture of the two strains.

Yeast one-hybrid (Y1H) assays were performed to confirm the regulatory relationship between PsAGL9 and *PsCYCD*. The results indicated that PsAGL9 interacted with the *PsCYCD* promoter (*proPsCYCD*-F) on SD/-Trp-Leu-His medium supplemented with 40 mM 3-amino-1, 2, 4-triazole (3-AT) (Fig. 4A)*.* Furthermore, the full-length *PsCYCD* promoter was divided into two segments based on the site of the CArG motif, and the results showed that the PsAGL9 protein could bind to the fragment containing the CArG motif (*proPsCYCD*-F1) (Fig. 4A). The GST-PsAGL9 fusion protein was purified, and electrophoretic mobility shift assay (EMSA) results showed strong binding signals after coincubation of GST-PsAGL9 and a biotin-labeled fragment (TTTGGCCAATTTTGGTTAA) containing the CArG motif, but these signals were weakened by the addition of 10× and 20× unlabeled probes, whereas the addition of a mutant probe did not affect the strength of the binding signal (Fig. 4B). To further validate the regulatory effect of PsAGL9 on *PsCYCD*, *35S::PsAGL9* and *proPsCYCD*-LUC were coinfiltrated into tobacco leaves. Dual-luciferase assays showed a significant increase in the fluorescence signal after cotransformation of *35S::PsAGL9* and *proPsCYCD*-LUC, which indicated that PsAGL9 could activate *PsCYCD* expression (Fig. 4C).

PsRGL1 interacted with PsAGL9 (Fig. 2), suggesting that PsAGL9 is an important component of the GA signaling pathway. To assess the impact of the interaction between PsAGL9 and PsRGL1 on the expression of *PsCYCD*, *35S::PsAGL9* + *proPsCYCD*-LUC and *35S::PsAGL9* + *35S::PsRGL1* + *proPsCYCD*-LUC combinations were infiltrated into tobacco leaves. Compared with the combination of *35S::PsAGL9* + *proPsCYCD*-LUC, a notably weaker fluorescence signal was observed for the combination of *35S::PsAGL9* + *35S::PsRGL1* + *proPsCYCD*-LUC, and the relative luciferase activity was consistent with the changes in the fluorescence signal (Fig. 4C). EMSAs demonstrated that PsAGL9 could bind to the *PsCYCD* promoter fragment containing CArG, and the intensity of the binding bands increased with increasing PsAGL9 protein content. However, the interaction between PsRGL1 and PsAGL9 inhibited the DNA-binding activity of PsAGL9 (Fig. 4D).

**Figure 5:**
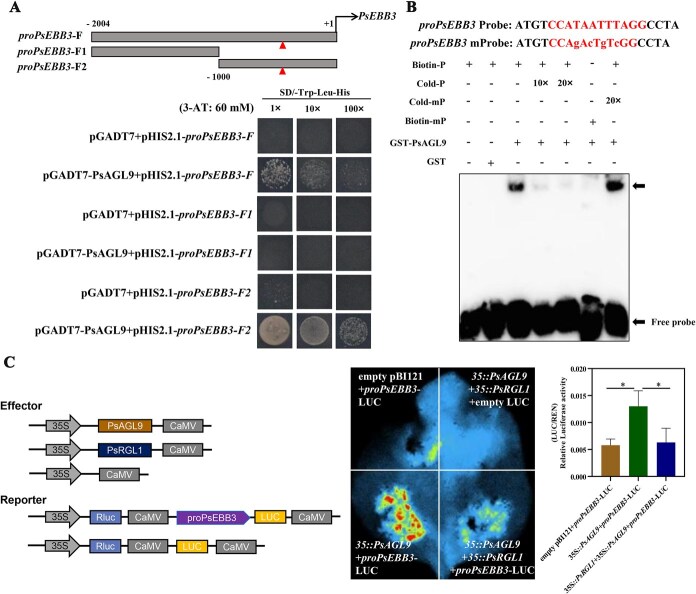
PsAGL9 directly bound and activated the expression of *PsEBB3.* A. PsAGL9 binding to a *PsEBB3* promoter fragment with the CArG motif using Y1H assays on SD/−Trp-Leu-His medium supplemented with 60 mM 3-AT. Diagram of *PsEBB3* promoter truncations; *proPsEBB3-F*, the full-length *PsEBB3* promoter; *proPsEBB3-F1* (−1001 to −2004 bp), a *PsEBB3* promoter fragment without the CArG motif; and *proPsEBB3-F2* (−1 to −1000 bp), a fragment with the CArG motif. The triangle marked the site of the CArG motif (CCATAATTTAGG). B. EMSAs revealed that PsAGL9 bound to the *PsEBB3* promoter fragment with the CArG motif. The the lowercase letters represented the mutant bases in the CArG motif. -, incubation without protein extract. 10× and 20× represented the addition of 10- and 20-fold concentrations, respectively, of the cold probe (cold-P) or mutant cold probe (cold-mP). C. Dual-luciferase assays showed that the interaction between PsAGL9 and PsRGL1 inhibited the expression of *PsEBB3. Agrobacterium* carrying the indicated combinations were injected into tobacco leaves, and the LUC images were captured for 72 h after injection. Relative luciferase activity was measured using the ratio of LUC to REN. Each value represented the mean of three biological replicates, and the vertical bars represented the mean ± SE. Asterisks represented statistically significant differences (^*^P < 0.05).

In addition, there was a nonclassical CArG motif (CCATAATTTAGG) at −451 to −462 of the 2004-bp *PsEBB3* promoter (Supplementary File 1), indicating that PsAGL9 might regulate *PsEBB3* expression. The Y1H results showed that PsAGL9 bound to the fragment with the CArG motif in the *PsEBB3* promoter (Fig. 5A), and EMSAs confirmed this binding (Fig. 5B). Dual-luciferase assays confirmed that PsAGL9 activated the expression of *PsEBB3* (Fig. 5C).

### Screening and identifying interaction proteins of PsAGL9

It is well known that MADS-box proteins usually form homo- or heterodimers to execute their functions [35]. For example, AGL9 and AGL15 recruit the FIS-PRC2 complex to trigger the transition from endosperm proliferation to embryo development in *Arabidopsis* [36]. Therefore, we employed pull-down assays coupled with LC–MS/MS to identify PsAGL9-interacting proteins. Three MADS-box proteins, including PsAGL9, were chosen for further investigation, suggesting that PsAGL9 might form homo- and heterodimers. After a local BLAST search with the tree peony genome and phylogenetic analysis (Figs S1 and S5), one MADS-box protein was first clustered with CsAGL6 (73.60% identity) and another was clustered with VvPI (75.83% identity). Therefore, they were named PsAGL6 and PsPI, respectively. The ORFs of *PsAGL6* and *PsPI* were 735 and 813 bp, encoding 245 and 271 amino acids, with molecular weights of 28.26 and 31.53 kDa, respectively (Genbank accession numbers: AXN75767.1 and QSB37188.1, respectively). The expression levels of *PsAGL6* and *PsPI* were significantly upregulated after chilling for 7 days, with an upward trend until 14 days. Additionally, they were induced by exogenous GA_3_ (Fig. S4). At the early stage of flowering, *PsPI* was highly expressed in petals and stamens, with lower expression levels in vegetative organs, such as roots, stems, leaves, and sepals. *PsAGL6* exhibited the highest expression level in sepals, followed by pistils and petals. Both genes showed relatively high expression levels in chilled buds (Fig. S3). These results suggested that they might function in the regulation of floral organ development and bud dormancy.

**Figure 6:**
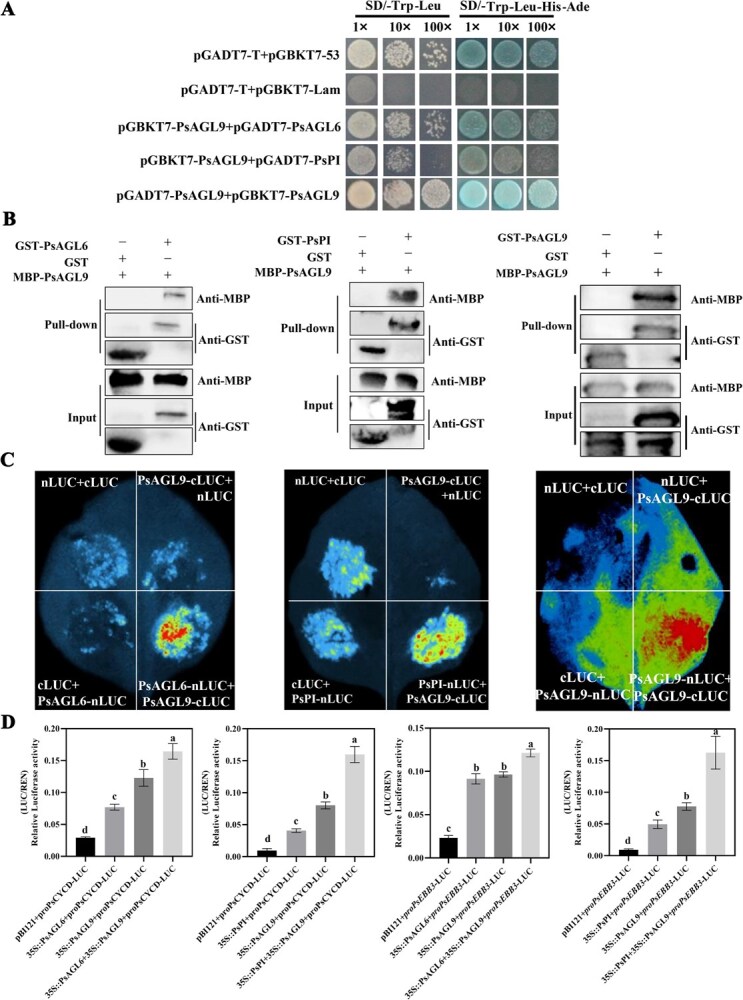
PsAGL9 interacting with PsAGL6 or PsPI to form homodimers and heterodimers. A. Y2H assays of the interaction of PsAGL9 with candidate proteins, including PsAGL6, PsPI, and PsAGL9, on SD/−Trp-Leu and SD/−Trp-Leu-His-Ade/X-a-Gal with 150 ng/ml AbA. The combination of pGADT7-T + pGBKT7-53 was used as a positive control, and pGADT7-T + pGBKT7-Lam served as a negative control. B. Pull-down assays. GST-PsAGL9, GST-PsAGL6, and GST-PsPI fusion proteins were coincubated with MBP-PsAGL9. After pull-down with GST beads, the eluates were analyzed using immunoblotting with anti-MBP or anti-GST antibodies. C. Luciferase complementary assays. PsAGL9-cLUC was cotransformed with nLUC-tagged PsAGL6, PsPI, and PsAGL9 into tobacco leaves, with the combination of nLUC + cLUC serving as a negative control. D. Dual-luciferase assays showed that the interactions between PsAGL9 and PsAGL6, PsAGL9, and PsPI further promoted the expression of *PsCYCD* and *PsEBB3*. *Agrobacterium* carrying the indicated combinations were injected into tobacco leaves, and the relative luciferase activity was determined using the ratio of LUC to REN. Each value represented the mean of at least three biological replicates, and the vertical bars represented the mean ± SE. Lowercase letters on the columns represented statistically significant differences (*P* < 0.05).

Y2H assays revealed that plants transformed with the combinations of pGBKT7-PsAGL9 + pGADT7-PsAGL6, pGBKT7-PsAGL9 + pGADT7-PsPI, and pGBKT7-PsAGL9 + pGADT7-PsAGL9 grew normally, suggesting that PsAGL9 interacted with itself, PsAGL6, or PsPI (Fig. 6A). Subsequently, GST-PsAGL9, GST-PsAGL6, and GST-PsPI fusion proteins were coincubated with MBP-PsAGL9, and a strong signal was detected in the subsequent pull-down assay. In luciferase complementation assays (LCAs), the combinations of PsAGL9-cLUC + PsAGL6-nLUC, PsAGL9-cLUC + PsPI-nLUC, and PsAGL9-cLUC + PsAGL6-nLUC exhibited stronger fluorescence signals than the controls. Overall, PsAGL9 could form a homodimer as well as heterodimers with PsAGL6 or PsPI (Fig. 6B and C).

Further, dual-luciferase assays were used to confirm the function of these interactions, and the results showed that heterodimers of PsAGL9 and PsAGL6 and PsAGL9 and PsPI further promoted the transcription of *PsCYCD* and *PsEBB3*. In addition, we found that both PsAGL6 and PsPI activated the expression of *PsCYCD* and *PsEBB3* (Fig. 6D). Y1H assays and EMSAs further verified that they could directly bind and activate target gene expression (Fig. S6).

## Discussion

Tree peony is known for its high ornamental value, and the transition from bud dormancy to dormancy release is a vital process for survival in winter and subsequent flowering in the following year. Activation of the GA pathway is necessary for dormancy release and budbreak in tree peony [14]. MADS-box genes play significant roles in the dormancy process in woody plants, including *Pyrus*, *Poplar*, *Prunus mume*, peach, and apple [20, 37–40]. In this study, we identified a MADS-box protein, PsAGL9, as a PsRGL1-interacting protein, and confirmed its involvement in the GA pathway. It was found to promote bud dormancy release by directly binding to and activating the expression of *PsCYCD* and *PsEBB3*. These findings revealed a new model of the GA pathway, namely, GA-PsRGL1-PsAGL9, for regulating bud dormancy release in tree peony.

### *PsAGL9* accelerates bud dormancy release in tree peony by regulating the cell cycle

MADS-box genes participate in plant growth and development, especially in regulating flowering time. For example, *SOC1* (*AGL20*) [41], *AGL15*, *AGL18* [42], *SVP* (*AGL22*) [43], *AGL24* [44], and *AGL6* [20] contribute to the regulation of plant floral development and flowering time. In the ABCDE model, E-class genes, including *AGL9* and *AGL2/3/4*, regulate flower organ development. *AGL9* overexpression accelerates early flowering and petal development [45, 46]. Recently, *DAM 1–6* have been identified as crucial regulators of dormancy transition [4, 20, 47, 48]. However, the specific roles of most MADS-box genes in bud dormancy release remain unclear. Mathiason *et al.* reported that prolonged chilling treatment contributes to the identification of dormancy-related genes in grape [49]. In this study, *PsAGL9* exhibited significantly high expression levels in various floral tissues at the early flowering stage, including petals, stamens, and pistils (Fig. S3), suggesting its involvement in floral development. Notably, *PsAGL9* transcript levels were upregulated following 7 days of chilling treatment, with the highest expression level after 21 days of chilling, indicating that it responded to both short-term and prolonged chilling exposures (Fig. 1). This highlights *PsAGL9* as an important dormancy-release-related gene in tree peony. Overexpression of *PsAGL9* confirmed that it facilitated bud dormancy release (Fig. 2). These results enriched our understanding of the role of MADS-box genes in regulating bud dormancy release in woody plants.

Although poplar and tree peony have different bud types, their bud dormancy has some similarities, as both belong to the endodormancy type. A recent study has shown that *EBB1* and *EBB3* act as positive regulators of bud dormancy release and budbreak in poplar [26]. In tree peony, *PsCYCD* is upregulated during chilling- and GA_3_-induced bud dormancy release [9, 30], which accelerates dormancy release and budbreak by activating cell proliferation [22]. Additionally, *PsEBB1* and *PsEBB3* are significantly upregulated by exogenous GA treatment [9]. Callose deposition blocks intercellular material transport channels in the dormant state [50, 51]. The subsequent dormancy release is characterized by the reactivation of these channels, facilitated by the action of beta-glucanase genes, *PsBG6* and *PsBG9*, in callose hydrolysis [52]. In the present study, *PsAGL9* overexpression increased the expression levels of *PsEBB1*, *PsEBB3*, *PsBG6*, *PsBG9*, and *PsCYCD*, implying that *PsAGL9* may facilitate bud dormancy release by modulating cellular proliferation or material transport. Promoter analysis indicated classical and nonclassical CArG motifs in the *PsCYCD* and *PsEBB3* promoters. Y1H assays, EMSAs, and luciferase assays further confirmed that PsAGL9 directly bound to and activated the expression of *PsCYCD* and *PsEBB3*. Based on our recent study, the marker gene *PsCYCD* is the same as *PsCYCD3.3* [22]. In poplar, EBB3 directly activates the expression of *CYCD3.1*, which promotes cell division within the apical meristem of the shoot [26]. Taken together, these data showed that *PsAGL9* promoted bud dormancy release mainly by restarting cell proliferation.

### *PsAGL9* participates in the GA pathway in the form of dimers to regulate bud dormancy release

GA signaling can intensively modulate the expression of *SOC1*, *FT*, *FLC*, and *LFY*, thereby accelerating flowering in plants [53]. Exogenous GAs significantly influence bud dormancy release, with many differentially expressed genes and proteins during this process being enriched in the GA signaling pathway [9, 54, 55]. DELLAs are key suppressors of the GA pathway. In Japanese apricot, exogenous GA induces the degradation of PmRGL2 and then activates NF-Y expression, thereby accelerating bud dormancy release [56]. Because DELLA proteins lack DNA-binding domains, they rely on binding proteins to regulate their target gene expression. Therefore, transcription factors interacting with DELLA have become focal points [57]. For example, DELLA proteins hinder the transition to flowering by interacting with FLC to enhance the transcriptional repression of *FT* and *SOC1* [58], and they also interact with WRKY75 to repress its activation ability [59]. Recent studies have implicated DELLA proteins in the control of dormancy release [12, 14]. We recently identified a key DELLA protein, PsRGL1, which participates in the GA signaling pathway and suppresses dormancy release in tree peony [14]. In this study, we identified proteins interacting with PsRGL1. Of particular interest was the identification of PsAGL9 as one of the interacting proteins, confirming its role as a novel component of the GA pathway in regulating bud dormancy release through direct interaction with PsRGL1. Further, EMSAs and dual-luciferase assays verified the actin model of this interaction and confirmed that high levels of PsRGL1 inhibited the binding of PsAGL9 to the *PsCYCD* promoter.

MADS-box proteins typically function as homodimers or heterodimers. For example, AGL9 and AGL15 recruit the FIS-PRC2 complex to initiate the transition from endosperm proliferation to embryo development in *Arabidopsis* [36]. The interaction between SOC1 and VRN1 influences the initiation of flowering in wheat [41]. In *Chrysanthemum*, ANR1 regulates lateral root development in the form of heterodimers with AGL21 in *Arabidopsis* [60]. Hence, screening for PsAGL9-interacting proteins helped expand the regulatory network involved in bud dormancy release in tree peony. In this study, three MADS-box proteins, PsAGL9, PsAGL6, and PsPI, were found to interact with PsAGL9 (Fig. 5). Further, we found that the interactions between PsAGL9 and PsAGL6 and between PsAGL9 and PsPI promoted the transcription of *PsCYCD* and *PsEBB3* (Fig. 6D), but the biological significance of homodimers requires further investigation. In the MADS-domain protein action model, the formation of quaternary complexes by these proteins requires the presence of two DNA-binding sites (CArG motifs) in the promoter region of the target gene [61]. In the present study, we only identified a unique CArG *cis*-element in the *PsCYCD* and *PsEBB3* promoters (Figs 4 and 5). This suggests that PsAGL9 might regulate bud dormancy break when present as dimers.

*PsAGL9*, *PsAGL6,* and *PsPI* were found to be upregulated by chilling, and their levels peaked after 14 days of chilling (Fig. 1, Fig. S3). PsAGL9 was abundant in the stamen and petal primordium from 7 to 14 days of chilling (Fig. 1). High levels of ubiquitination of PsRGL1 occurred ~21 days of chilling. These findings suggested that PsAGL9 formed heterodimers in the dormant buds at 14–21 days of chilling, during the transition stage from endodormancy to endodormancy release. However, the functions of the interacting proteins required further investigation.

## Conclusions

**Figure 7:**
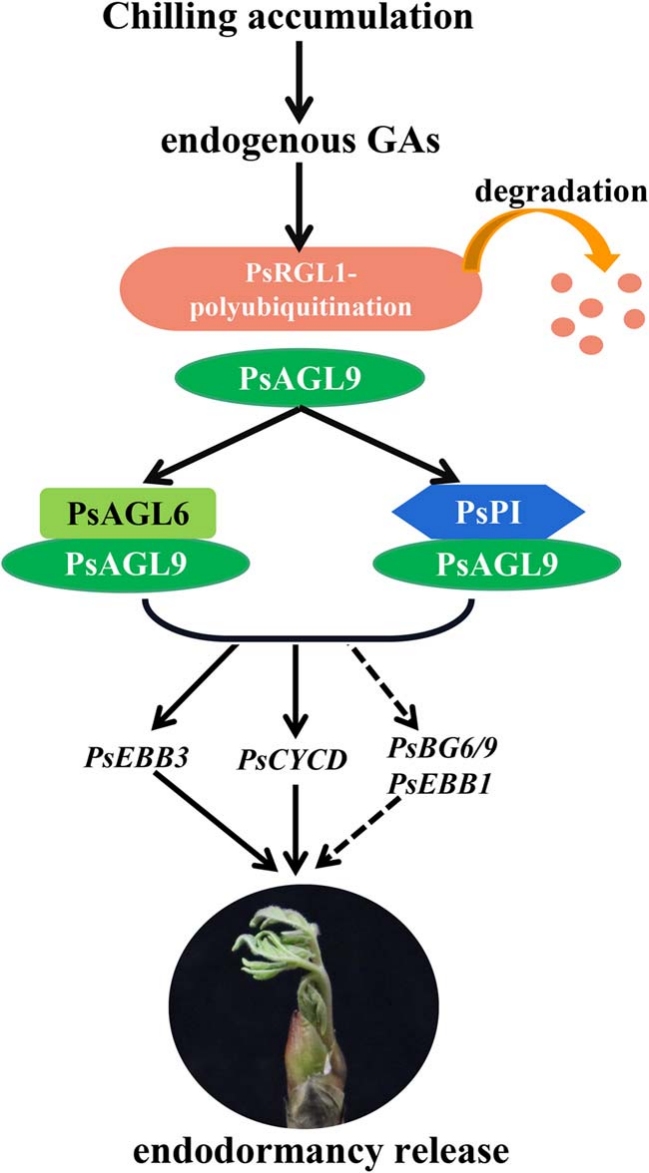
A model of the facilitation of endodormancy release by PsAGL9 in tree peony. At the early stage of bud dormancy, *PsAGL9* is rapidly induced by chilling and exogenous GA_3_, and is then bound by PsRGL1. This interaction prevents PsAGL9 from binding to the promoters of downstream genes. Prolonged chilling time promotes the degradation of PsRGL1, resulting in the release of PsAGL9. Subsequently, PsAGL9 forms heterodimers with PsAGL6 or PsPI. These heterodimers directly activate the expression of *PsCYCD* and *PsEBB3*, thereby accelerating dormancy release by initiating cell proliferation in tree peony.

Our findings present a model whereby *PsAGL9* facilitates dormancy break in tree peony (Fig. 7). In the early stage of bud dormancy, *PsAGL9* is rapidly induced by chilling and exogenous GA_3_, and is captured by abundant PsRGL1. This interaction prevents PsAGL9 from binding to the promoters of its target genes. With prolonged chilling, the increasing endogenous bioactive GAs promote the degradation of PsRGL1, resulting in the release of PsAGL9 and the formation of heterodimers with PsAGL6 or PsPI. These heterodimers directly bind to the promoters of *PsCYCD* and *PsEBB3* and activate their expression, thereby accelerating dormancy release by promoting cell proliferation in tree peony.

## Materials and methods

### Plant materials and growth conditions

Four-year-old tree peony variety ‘Luhehong’ specimens, originating from the tree peony garden of Qingdao Agricultural University, were potted and transferred into refrigerated housing (4°C, 24 h in darkness) when the daily average temperature fell <10°C. Apical buds were collected every 7 days up to 28 days after chilling treatment.

After 7 days of chilling treatment, plants in another group were moved to a phytotron (22°C, 16 h of light/8 h of darkness), and then treated with 200 mg/l of GA_3_ according to the method described by Zhang *et al.* [9]. Apical buds were collected at 0, 12, 24, 48, and 72 h. All collected buds were immediately placed in liquid nitrogen and preserved at −80°C.

### Bioinformatic analysis

Tree peony AGL9, AGL6, PI, and corresponding proteins in other known plants were aligned using ClustalW. A phylogenetic tree was constructed using the neighbor-joining method in MEGA-X. Subcellular localization was predicted using the online tool WoLF PSORT.

### qRT-PCR

Total RNA was extracted using the RNAprep Plant Kit (GBTbiotech, Beijing, China), and first-strand cDNA was synthesized using SuperScript IV (Thermo Fisher, Waltham, MA, USA) according to the manufacturer’s protocol. Two microliters of cDNA served as the template for PCR using the SYBR® Premix Ex TaqTM II Kit, following the manufacturer’s instructions (Accurate Biotechnology, Hunan, China). Relative expression levels were calculated according to the 2^-ΔΔCt^ method. The primers used for qRT-PCR were listed in Supplementary Table 1.

### *In situ* hybridization

Apical buds were collected after chilling treatment for 0–21 days, preserved in a solution of 3.7% formalin-acetic acid-alcohol, and then dehydrated with a series of ethanol concentrations before being embedded in paraffin. Probes for *PsAGL9* were generated using PCR amplification with primers designed to contain binding sites for T7 and SP6 RNA polymerases. *In situ* hybridization was performed using a DIG RNA Labeling Kit (SP6/T7) (Roche, Basel, Switzerland) according to the manufacturer’s instructions. The primer pairs used for *in situ* hybridization were listed in Supplementary Table 1.

### Subcellular localization

The coding sequence of *PsAGL9*, without the stop codon, was inserted into the pCAMBIAsuper1300-GFP vector to generate a *PsAGL9*-GFP fusion construct, which was used to transform competent cells of *Agrobacterium tumefaciens* strain GV3101, with an empty GFP plasmid serving as a control. Tobacco leaves were infiltrated following the method described by Gao *et al.* [14], and after infiltration for 48 h, green fluorescence was detected using a confocal laser scanning microscope (TCS SP5II; Agilent, Santa Clara, CA, USA). DAPI staining was applied to visualize the nuclei. The primers were listed in Supplementary Table 1.

### Pull-down assay coupled with LC–MS/MS

After chilling treatment, the buds were ground, and total protein was extracted using an extraction buffer comprising 50 mM Tris–HCl (pH 7.5), 150 mM NaCl, 10 mM MgCl_2_, 10 μM MG132, 1 mM PMSF, 0.1% NP-40, and 1× Protease Inhibitor Cocktail (Roche). *PsRGL1* and *PsAGL9* ORFs were inserted into the pGEX-4 T-1 (GST) vector. GST protein and GST-PsRGL1 and GST-PsAGL9 fusion proteins were purified and then immobilized with Glutathione Magnetic Agarose Beads (MedChemExpress, Monmouth Junction, NJ, USA). Total proteins were incubated with immobilized GST, GST-PsRGL1, and GST-PsAGL9 proteins for 1 h at 4°C. Protein-bound beads were cleaned three times using 1 ml of phosphate-buffered saline and then analyzed using a pull-down assay coupled with LC–MS/MS by Bioprofile (Shanghai, China). Three independent replicates were analyzed.

### Y2H assay

The ORFs of *PsAGL9*, *PsAGL6*, and *PsPI* were introduced separately into the pGADT7 vector. The *PsAGL9* ORF and the *PsRGL1* coding sequence, lacking the DELLA domain, were cloned into the pGBKT7 vector and transformed into Y2HGold yeast competent cells (Clontech, Palo Alto, NJ, USA). Protein interactions were identified using a Matchmaker Gold Yeast Two-Hybrid assay kit (Clontech, Palo Alto, NJ, USA), following the manufacturer’s protocol. The primers used in Y2H assays were listed in Supplementary Table 1.

### Pull-down assay

The *PsAGL9* ORF was cloned into the pMAL-c5x (MBP) vector, and the ORFs of *PsRGL1*, *PsPI*, *PsAGL6*, and *PsAGL9* were cloned into the GST vector. GST-PsRGL1, GST-PsAGL6, GST-PsPI, GST-PsAGL9, and MBP-PsAGL9 fusion proteins were then purified. The soluble GST fusion proteins were extracted and immobilized on Glutathione Magnetic Agarose Beads (MedChemExpress). The MBP-PsAGL9 fusion protein was then incubated with these beads. Proteins adhering to the beads were separated using sodium dodecyl sulfate-polyacrylamide gel electrophoresis and were subsequently characterized using anti-MBP or anti-GST antibodies (ABclonal, Wuhan, China). The primers used for pull-down assays were listed in Supplementary Table 1.

### Luciferase complementation assay

The ORFs of *PsRGL1*, *PsAGL6*, *PsAGL9*, and *PsPI* were inserted into the pCAMBIA1300-nLUC (nLUC) vector, and the ORF of *PsAGL9* was cloned into the pCAMBIA1300-cLUC (cLUC) vector. Tobacco leaves were inoculated with *Agrobacterium* strain GV3101 carrying recombinant plasmids. After 3 days, D-luciferin (1 mM) was sprayed onto the leaves, which were then kept in the dark for 10 min, followed by imaging using a low-light CCD imaging system (Newton 7.0; Vilber, Paris, France) to capture luminescent signals. Three independent replicates were analyzed. The primers used for the LCAs were listed in Supplementary Table 1.

### *PsAGL9* overexpression

The ORF of *PsAGL9* was cloned into the pBI121 vector controlled by the cauliflower mosaic virus 35S (*CaMV 35S*) promoter and then transformed into *A. tumefaciens* EHA105, which was used to transform 10-day-chilled buds at 0.3 MPa for 3–4 min using the vacuum permeation method described by Gao *et al.* [14]. The buds were then grafted as scions to the roots of 3-year-old red peony (*Paeonia lactiflora*) plants. After 3 days of culture in the dark, they were transferred into a greenhouse (16 h light/8 h dark, 22°C), with plants transformed using an empty pBI121 vector serving as controls. qRT-PCR was used to screen for positive transgenic *PsAGL9* buds at 7 DAI. Relative expression levels of the marker genes *PsCYCD*, *PsBG6*, *PsBG9*, *PsEBB1*, and *PsEBB3* were determined in the *PsAGL9-*OE buds with high transformation efficiency. The budbreak rate was also recorded daily, with statistical significance set at *P* < 0.05. The primer sequences for *PsAGL9-*OE vector and qRT-PCR analysis were listed in Supplementary Table 1.

### Y1H assay

The *PsAGL9* ORF was cloned into the pGADT7 vector. The *PsCYCD* promoter (*proPsCYCD-F*) fragment was divided into two segments based on the CArG site, namely, *proPsCYCD-F1* containing the CArG motif, and *proPsCYCD-F2* without the CArG motif. They were subsequently cloned into the pHIS2.1 vector. The combinations of pGADT7-*PsAGL9 +* pHIS2.1-*proPsCYCD-F*, pGADT7-*PsAGL9 +* pHIS2.1-*proPsCYCD-F1*, and pGADT7-*PsAGL9 +* pHIS2.1-*proPsCYCD-F2* were cotransformed into yeast Y187 competent cells (Clontech, Palo Alto, NJ, USA), while the combinations of pGADT7 + pHIS2.1-*proPsCYCD-F*, pGADT7 + pHIS2.1-*proPsCYCD-F1*, and pGADT7 + pHIS2.1-*proPsCYCD-F2* served as controls. Yeast transformants were cultured on SD/−Trp-Leu-His medium supplemented with 3-AT. The Y1H assay was also used to determine PsAGL9 binding to the *PsEBB3* promoter using a method similar to the one described above. The primers used for Y1H assays were listed in Supplementary Table 1.

### EMSA

The ORF of *PsAGL9* was inserted into the pGEX-4 T-1 vector and then transformed into *Escherichia coli* BL21 competent cells. Isopropyl β-D-1-thiogalactopyranoside (0.1 mM) was used to induce fusion protein expression. Fragments of the *PsCYCD* and *PsEBB3* promoters were synthesized and labeled with biotin. Unlabeled fragments of the same sequence or corresponding sequences with mutated CArG motifs were used as competitors. The binding of the PsAGL9 protein to target gene promoters was detected using a Chemiluminescent EMSA Kit (Beyotime, China) following the manufacturer’s instructions.

*PsAGL9* and *PsRGL1* ORFs were cloned into the pCAMBIAsuper1300-GFP and pCXSN-HA vectors, respectively. Tobacco leaves were infiltrated with *Agrobacterium* strains containing PsAGL9-GFP or HA-PsRGL1 constructs alone or as a 1:1 mixture. Protein extracts from these leaves were used to analyze the abundance of PsRGL1 and PsAGL9 proteins using anti-HA and anti-GFP antibodies, based on the method described by De Lucas *et al.* [62]. EMSAs was performed as previously described. A fragment of the *PsCYCD* promoter containing the CArG motif (TTTGGCCAATTTTGGTTAAA) was used for retardation analysis. The primers used for EMSAs were listed in Supplementary Table 1.

### Dual-luciferase assays

*PsCYCD* and *PsEBB3* promoter sequences were inserted into the pGreen II 0800-LUC vector. The coding sequences of *PsRGL1* and *PsAGL9* were cloned into the pBI121 vector driven by the *CaMV 35S* promoter and transformed into *Agrobacterium* strain GV3101 (pSoup). Tobacco leaves were then infected as previously described [22]. To confirm the function of the interactions between PsAGL9 and PsAGL6 or PsAGL9 and PsPI, the ORFs of *PsAGL6* and *PsPI* were cloned into the pBI121 vector, and the combinations, including *35S::PsAGL9* + *35S::PsAGL6* + *proPsCYCD*-LUC (*proPsEBB3*-LUC), *35S::PsAGL9* + *35S::PsPI* + *proPsCYCD*-LUC (*proPsEBB3*-LUC), *35S::PsAGL9* + *proPsCYCD*-LUC (*proPsEBB3*-LUC), *35S::PsAGL6* + *proPsCYCD*-LUC (*proPsEBB3*-LUC), *35S::PsPI* + *proPsCYCD*-LUC (*proPsEBB3*-LUC), and empty pBI121 + *proPsCYCD*-LUC (*proPsEBB3*-LUC), were cotransformed into tobacco leaves. After 1 day of culture in the dark at 22°C and 3 days under LD conditions (16 h light/8 h), D-luciferin (1 mM) was sprayed onto the leaves, which were subsequently imaged using a low-light CCD imaging apparatus (Newton 7.0) to capture the luminescence signal. Relative luciferase activity was calculated. Each data point represented a minimum of three replicates, and each assay was repeated three to five times independently. The primers used for dual-luciferase assays were listed in Supplementary Table 1.

## Supplementary Material

Web_Material_uhaf043

## Data Availability

The accession numbers of genes used in this manuscript were as following: PsEBB1 (OP095871), PsEBB3 (OP095872), PsCYCD (OP095873), PsBG6 (OP095874), and PsBG9 (OP734236).
