# Supplementary material for: MADS-domain transcription factor AGAMOUS LIKE-9 participates in the gibberellin pathway to promote bud dormancy release of tree peony
Source: Hortic Res. 2025 Feb 10;12(5):uhaf043. doi: 10.1093/hr/uhaf043 (PMC11997425; doi:10.1093/hr/uhaf043)
Supplement: Web_Material_uhaf043 [file web_material_uhaf043.zip › Supplementary figures.pdf]

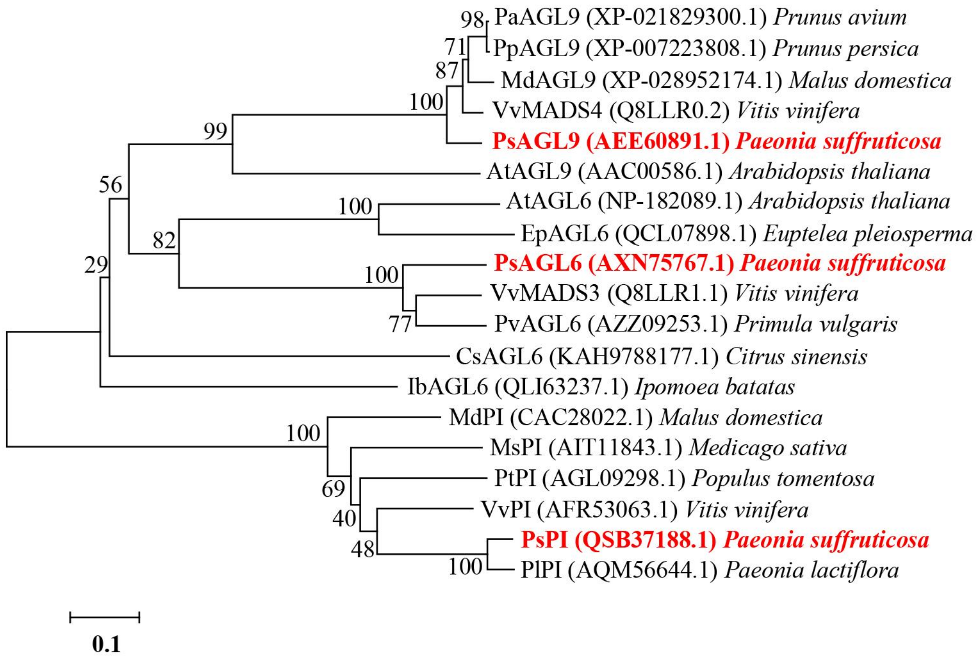

Supplementary figure 1 Phylogenetic analysis of PsAGL9, PsAGL6 and PsPI using maximum likelihood constructed with MEGA-X. Bootstrap values were 1000 replicates. Red arrow marked the AGL9, AGL6, PI protein of tree peony, and the Genbank accession numbers of the corresponding proteins were in brackets. Ps, *Paeonia suffruticosa*; Vv, *Vitis vinifera*; Md, *Malus domestica*; Pp, *Prunus persica*; Pa, *Prunus avium*; Pt, *Populus tomentosa*; At, *Arabidopsis thaliana*; Ep, *Euptelea pleiosperma*; Pv, *Primula vulgaris*; Cs, *Citrus sinensis*; Ib, *Ipomoea batatas*; MsPI, *Medicago sativa*; Pi, *Paeonia lactiflora.*

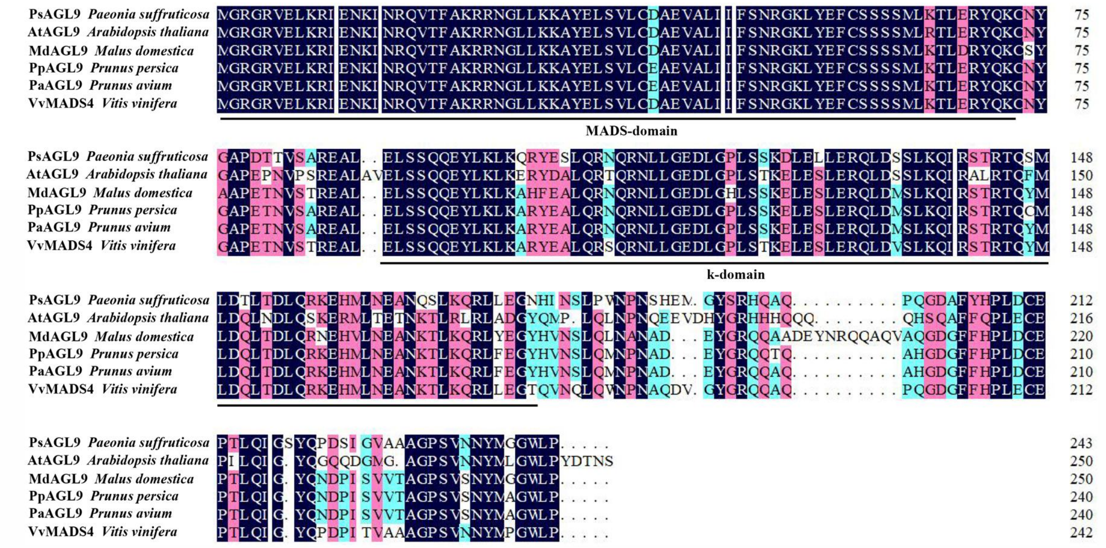

Supplementary figure 2 Multiple alignment of PsAGL9 and the known AGL9 proteins in other plants using ClustalW. The MADS- and K-domains were underlined. Different colors indicated different homology. Ps, *Paeonia suffruticosa*; Vv, *Vitis vinifera*; Md, *Malus domestica*; Pp, *Prunus persica*; At, *Arabidopsis thaliana*; Pa, *Prunus avium*. Navy blue represented sites with the same amino acid, pink marked high homogenous sites, and blue and white represented sites of different amino acids.

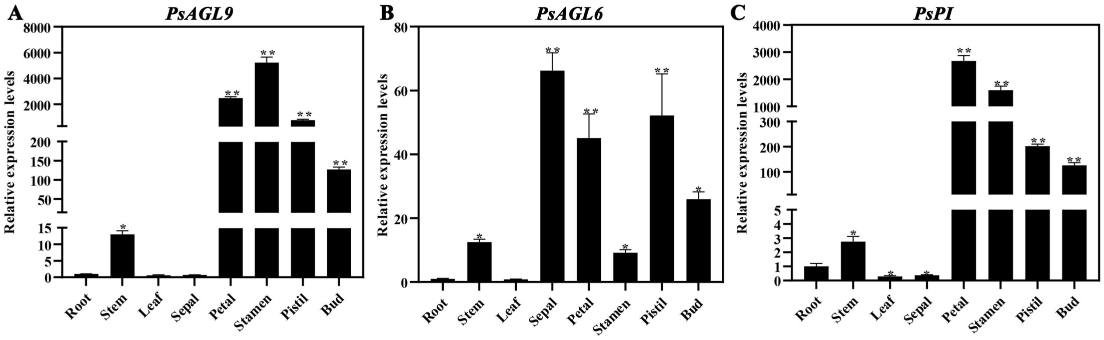

Supplementary figure 3 Expression patterns of *PsAGL9*, *PsAGL6*, and *PsPI* at the early stage of flowering and the mixed buds of different chilling days using qRT-PCR. Error bars for all data represent the error value using three replicates, and asterisks represent statistically significant differences (* $p < 0.05$; ** $p < 0.01$).

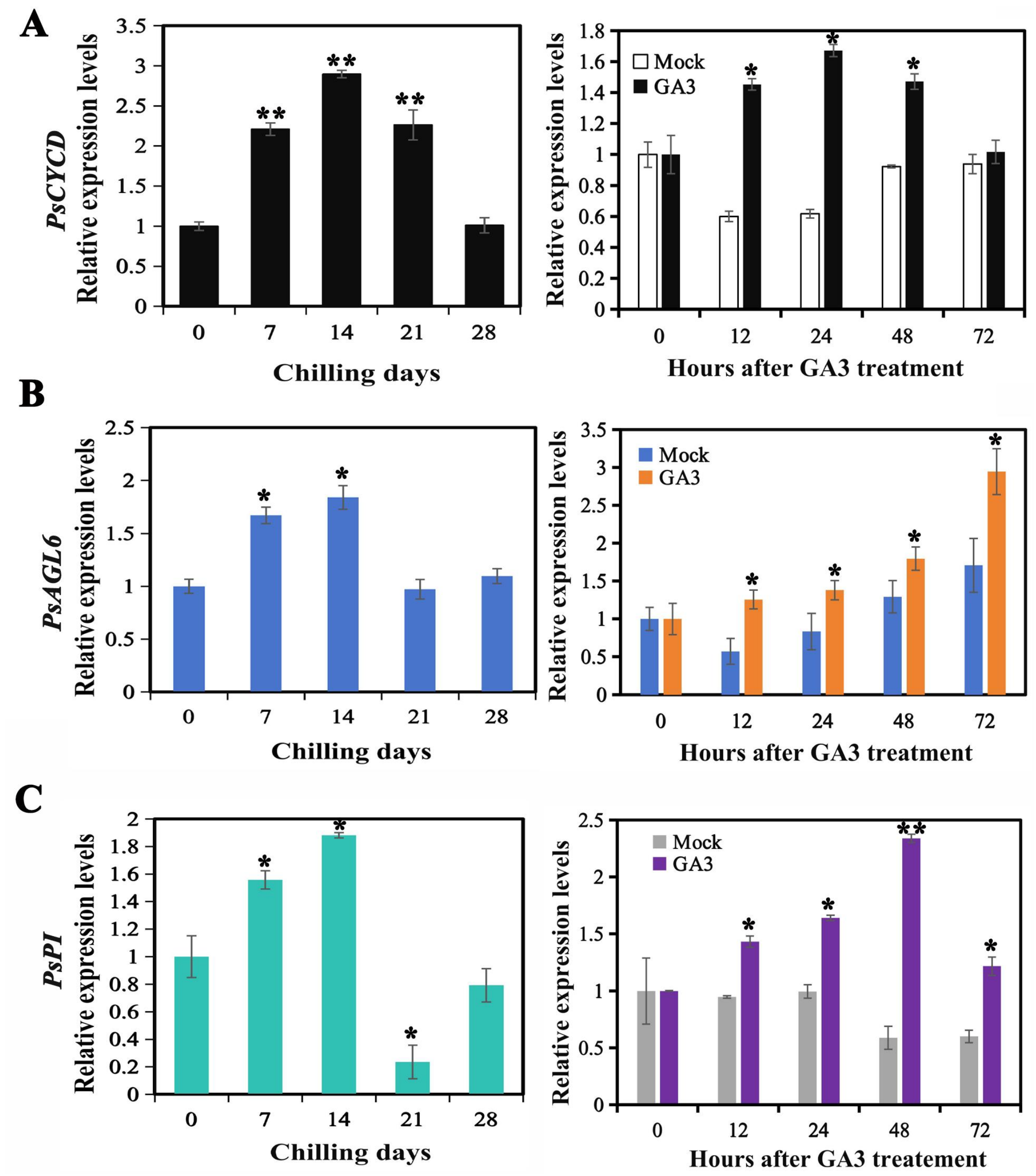

Supplementary figure 4 Expressions of *PsCYCD* during chilling- (A) and exogenous GA_3-induced (B) bud dormancy release. Error bars for all data represent the error value using three replicates, and asterisks represent statistically significant differences (* $p < 0.05$; ** $p < 0.01$).

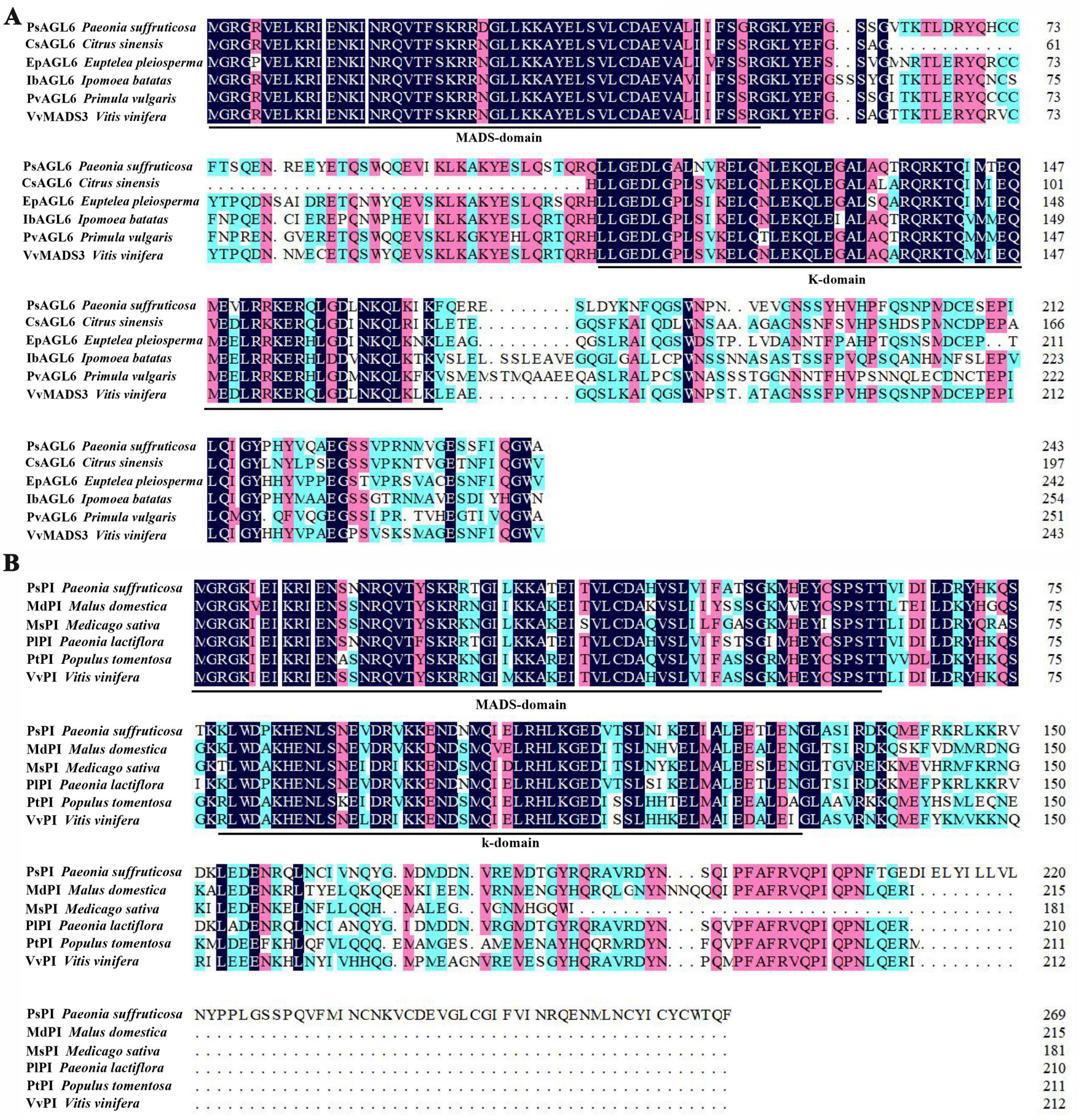

Supplementary figure 5 Multiple alignment of PsAGL6, PsPI and the known AGL9, PI proteins in other plants using ClustalW. The MADS- and K-domains were underlined. Different colors indicated different homology. Ps, *Paeonia suffruticosa*; Cs, *Citrus sinensis*; Ib, *Ipomoea batatas*; Ep, *Euptelea pleiosperma*; Pv, *Primula vulgaris*; Vv, *Vitis vinifera*; Md, *Malus domestica*; MsPI, *Medicago sativa*; Pl, *Paeonia lactiflora*; Pt, *Populus tomentosa*. Navy blue represented sites with the same amino acid, pink marked high homogenous sites, and blue and white represented sites of different amino acids.

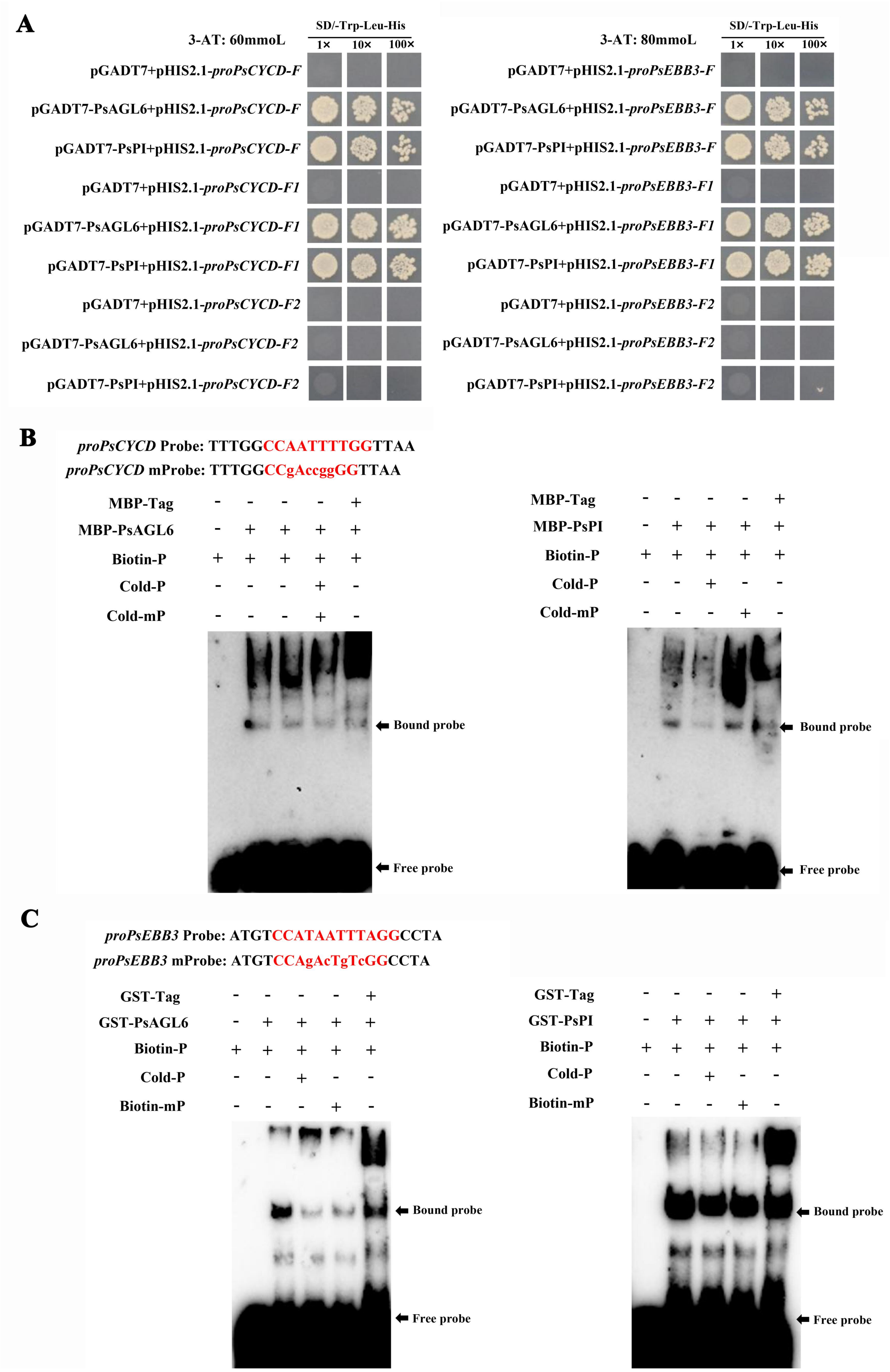

Supplementary figure 6 PsAGL6 and PsPI directly bound the promoters of *PsCYCD* and *PsEBB3*. A. PsAGL6 and PsPI bound the fragment of *PsCYCD* and *PsEBB3* promoters

containing CArG motif as shown by yeast one-hybird (Y1H) assays on SD/-Trp-Leu-His medium supplemented with 3-AT. *proPsCYCD-F*, full length of *PsCYCD* promoter; *proPsCYCD-F1*, the fragment of *PsCYCD* promoter containing CArG motif; *proPsCYCD-F2*, the fragment of *PsCYCD* promoter without CArG motif. B. Electrophoretic mobility shift assay (EMSA) showed PsAGL6 and PsPI binding to the *PsCYCD* promoter fragment with CArG motif. C. PsAGL6 and PsPI bound to the *PsEBB3* promoter fragment with CArG motif using EMSA assay. Red font marked CArG motif, and the lowercase letters represented the mutant bases in CArG motif. -, incubation without protein extract.
