# Supplementary material for: MADS-domain transcription factor AGAMOUS LIKE-9 participates in the gibberellin pathway to promote bud dormancy release of tree peony
Source: Hortic Res. 2025 Feb 10;12(5):uhaf043. doi: 10.1093/hr/uhaf043 (PMC11997425; doi:10.1093/hr/uhaf043)
Supplement: Web_Material_uhaf043 [file web_material_uhaf043.zip › Supplementary Table 1 The primer information used20250120.docx]

**Supplementary Table 1 The primer information used in this study**

| **Name** | **Sequence (5’-3’)** | **purpose** |
| --- | --- | --- |
| PsAGL9-real-F | AGGACCTGGAGCTACTCGAA | qRT-PCR |
| PsAGL9-real-R | AAAGGCATCACCCTGTGGTT |  |
| PsAGL6-real-F | CAGAGCTGGCAACAAGAGGTA |  |
| PsAGL6-real-R | GCTGCTTGTTTAAGTCGCCA |  |
| PsPI-real-F | CAAGCATTCGGGACAAGCAG |  |
| PsPI-real-R | GATTGGCTGCACACGAAAGG |  |
| PsActin-real-F | GAGAGATTCCGTTGCCCTGA |  |
| PsActin-real-R | CTCAGGAGGAGCAACCACC |  |
| PsCYCD-real-F | GAGGCCGTGGATTGGATTCT |  |
| PsCYCD-real-R | AAAAGGGGCACTTGGGTCTC |  |
| PsEBB1-real-F | AATAGCCCGCGAAGTCCAAA |  |
| PsEBB1-real-R | GGGATCTGATGAACCAGCCC |  |
| PsEBB3-real-F | GGTGAGATTACTCCGCCACC |  |
| PsEBB3-real-R | CGACCCTGAATCTGAGACCG |  |
| PsBG6-real-F | CCTACTACCCGGCCACAAAG |  |
| PsBG6-real-R | CTACTGAAAGCACCCGCAGA |  |
| PsBG9-real-F | TCATCTTCGCTCCGATGCTC |  |
| PsBG9-real-R | TCGGCCACAACTATGTCCAC |  |
| PsAGL9-GFP-F | atacaccaaatcgactctagaATGGGGAGGGGTAGGGTTG  (*Xba*I) | Subcellular localization |
| PsAGL9-GFP-R | gcccttgctcaccatggtaccTGGTAACCATCCCCCCATG  (*Kpn*I) |  |
| PsAGL9-LP-F | GAATTGTAATACGACTCACTATAGGGAATTGAGAACAAGATCAAC | In situ hybridization |
| PsAGL9-LP-R | CATAACGTTG TTTCAGCT |  |
| PsAGL9-RP-F | AATTGAGAACAAGATCAAC |  |
| PsAGL9-RP-R | GAATTGTAATACGACTCACTATAGGGCATAACGTTG TTTCAGCT |  |
| pBI121-PsAGL9-F | acgggggactctagaggatccATGGGGAGGGGTAGGGTTG  (*Bam*HI) | *PsAGL9* over-expression |
| pBI121-PsAGL9-R | ataagggactgaccacccgggTCATGGTAACCATCCCCCCA  (*Sma*I) |  |
| PsRGL1-BD-F | atggccatggaggccgaattcATGGCCACCTACGACCCTG  (*Eco*RI) | yeast two-hybrid assays |
| PsRGL1-BD-R | ccgctgcaggtcgacggatccTCACAACACATTATGATTTATTATTCTACTTG  (*Bam*HI) |  |
| PsAGL9-AD-F | gccatggaggccagtgaattcATGGGGAGGGGTAGGGTTG  (*Eco*RI) |  |
| PsAGL9-AD-R | cagctcgagctcgatggatccTGGTAACCATCCCCCCATG  (*Bam*HI) |  |
| PsAGL6-AD-F | gccatggaggccagtgaattcATGGGGAGAGGAAGGGTAGAGC  (*Eco*RI) |  |
| PsAGL6-AD-R | cagctcgagctcgatggatccAAGGGCCCATCCTTGGATG  (*Bam*HI) |  |
| PsPI-AD-F | gccatggaggccagtgaattcATGGGAAGAGGTAAGATTGAGATCA  (*Eco*RI) |  |
| PsPI-AD-R | cagctcgagctcgatggatccATAAAATTGAGTCCAACAATAACAAATATAA  (*Bam*HI) |  |
| PsAGL9-BD-F | atggccatggaggccgaattcATGGGGAGGGGTAGGGTTG  (*Eco*RI) |  |
| PsAGL9-BD-R | ccgctgcaggtcgacggatccTGGTAACCATCCCCCCATG  (*Bam*HI) |  |
| PsAGL6-nLUC-F | acgggggacgagctcggtaccATGGGGAGAGGAAGGGTAGAGC  (*Kpn*I) | LCI |
| PsAGL6-nLUC-R | tgtagtccatttgttggatccAAGGGCCCATCCTTGGATG  (*Bam*HI) |  |
| PsPI-nLUC-F | acgggggacgagctcggtaccATGGGAAGAGGTAAGATTGAGATCA  (*Kpn*I) |  |
| PsPI-nLUC-R | tgtagtccatttgttggatccATAAAATTGAGTCCAACAATAACAAATATAA  (*Bam*HI) |  |
| PsRGL1-nLUC-F | acgggggacgagctcggtaccATGGCCACCTACGACCCTG  (*Kpn*I) |  |
| PsRGL1-nLUC-R | tgtagtccatttgttggatccCAACACATTATGATTTATTATTCTACTTGCA  (*Bam*HI) |  |
| PsAGL9-cLUC-F | tacgcgtcccggggcggtaccATGGGGAGGGGTAGGGTTG  (*Kpn*I) |  |
| PsAGL9-cLUC-R | tgtagtccatttgttggatccTCATGGTAACCATCCCCCCA  (*Bam*HI) |  |
| PsAGL9-nLUC-F | acgggggacgagctcggtaccATGGGGAGGGGTAGGGTTG  (*Kpn*I) |  |
| PsAGL9-nLUC-R | tgtagtccatttgttggatccTGGTAACCATCCCCCCATG  (*Bam*HI) |  |
| GST-PsRGL1-F | ccgcgtggatccccggaattcATGGCCACCTACGACCCTG  (*Eco*RI) | Fusion protein expression |
| GST-PsRGL1-R | gtcacgatgcggccgctcgagTCACAACACATTATGATTTATTATTCTACTTG  (*Xho*I) |  |
| MBP-PsAGL9-F | gagggaaggatttcacatatgATGGGGAGGGGTAGGGTTG  (*Nde*I) |  |
| MBP-PsAGL9-R | ttaattacctgcagggaattcTCATGGTAACCATCCCCCCA  (*Eco*RI) |  |
| GST-PsAGL9-F | ccgcgtggatccccggaattcATGGGGAGGGGTAGGGTTG  (*Eco*RI) |  |
| GST-PsAGL9-R | gtcacgatgcggccgctcgagTCATGGTAACCATCCCCCCA  (*Xho*I) |  |
| MBP-PsAGL6-F | gagggaaggatttcacatatgATGGGGAGAGGAAGGGTAGAGC  (*Nde*I) |  |
| MBP-PsAGL6-R | ttaattacctgcagggaattcTCAAAGGGCCCATCCTTGG  (*Eco*RI) |  |
| GST-PsAGL6-F | ccgcgtggatccccggaattcATGGGGAGAGGAAGGGTAGAGC  (*Eco*RI) |  |
| GST-PsAGL6-R | gtcacgatgcggccgctcgagTCAAAGGGCCCATCCTTGG  (*Xho*I) |  |
| MBP-PsPI-F | gagggaaggatttcacatatgATGGGAAGAGGTAAGATTGAGATCA  (*Nde*I) |  |
| MBP-PsPI-R | ttaattacctgcagggaattcTCAATAAAATTGAGTCCAACAATAACAA  (*Eco*RI) |  |
| GST-PsPI-F | ccgcgtggatccccggaattcATGGGAAGAGGTAAGATTGAGATCA  (*Eco*RI) |  |
| GST-PsPI-R | gtcacgatgcggccgctcgagTCAATAAAATTGAGTCCAACAATAACAA  (*Xho*I) |  |
| HA-PsRGL1-F | ccaatacttgtatgggtcgacATGGCCACCTACGACCCTG  (SalI) |  |
| HA-PsRGL1-R | aatcaggaagggatggctgaggTCACAACACATTATGATTTATTATTCTACTTG  (*Bbv*cI) |  |
| pHIS2.1-*proPsCYCD*-F | gactcactatagggcgaattcCGATGATTTCTGTCGATAAAGACAG  (*Eco*RI) | yeast one-hybrid assays |
| pHIS2.1-*proPsCYC*D-R | attactagtggatccacgcgtCTTCTTCTGAGAAGAAGAGACCAAAGC  (*Mlu*I) |  |
| pHIS2.1-*proPsCYCD*-F1 | gactcactatagggcgaattcCGATGATTTCTGTCGATAAAGACAG  (*Eco*RI) |  |
| pHIS2.1-proPsCYCD-R1 | attactagtggatccacgcgtGCCTCAGTGAGGCCATGAATC  (*MIu*I) |  |
| pHIS2.1-proPsCYCD-F2 | gactcactatagggcgaattcCACGGCCTCATATGATGTGGC  (*Eco*RI) |  |
| pHIS2.1-*proPsCYCD*-R2 | attactagtggatccacgcgtCTTCTTCTGAGAAGAAGAGACCAAAGC  (*MIu*I) |  |
| pHIS2.1-*proPsEBB3*-F | gactcactatagggcgaattcTTGAATTTGATTACTTTAAAATTACTTGTTT  (*Eco*RI) |  |
| pHIS2.1-*proPsEBB3*-R | attactagtggatccacgcgtGGCGTCGTTTTGGGCCCA  (*Mlu*I) |  |
| pHIS2.1-*proPsEBB3*-F1 | gactcactatagggcgaattcTTGAATTTGATTACTTTAAAATTACTTGTTT  (*Eco*RI) |  |
| pHIS2.1-*proPsEBB3*-R1 | attactagtggatccacgcgtTCTCCTACGTTTCCAAGTTTTTGTT  (*Mlu*I) |  |
| pHIS2.1-*proPsEBB3*-F2 | gactcactatagggcgaattcTTCTATTAACATTTTCTTTTAGAAAGTAGAGG  (*Eco*RI) |  |
| pHIS2.1-*proPsEBB3*-R2 | attactagtggatccacgcgtGGCGTCGTTTTGGGCCCA  (*Mlu*I) |  |
| *proPsCYCD*-LUC-F | gtcgacggtatcgataagcttCGATGATTTCTGTCGATAAAGACAG  (*Hin*d Ш) | dual luciferase assays |
| *proPsCYCD*-LUC-R | ggcggccgctctagaactagtCTTCTTCTGAGAAGAAGAGACCAAAGC  (*Spe*I) |  |
| *ProPsEBB3*-LUC-F | gtcgacggtatcgataagcttGAGTCGAAGAAGAAACAAAAACAAAA  (*Hin*d Ш) |  |
| *ProPsEBB3*-LUC-R | cgctctagaactagtggatccGGCGTCGTTTTGGGCCCA  (*Bam*HI) |  |
| pBI121-PsRGL1-F | acgggggactctagaggatccATGGCCACCTACGACCCTG  (*Bam*HI) |  |
| pBI121-PsRGL1-R | ataagggactgaccacccgggTCACAACACATTATGATTTATTATTCTACTTG  (*Sma*I) |  |
| pBI121-PsAGL6-F | acgggggactctagaggatccATGGGGAGAGGAAGGGTAGAGC  (*Bam*HI) |  |
| pBI121-PsAGL6-F | ataagggactgaccacccgggTCAAAGGGCCCATCCTTGG  (*Sma*I) |  |
| pBI121-PsPI-F | acgggggactctagaggatccATGGGAAGAGGTAAGATTGAGATCA  (*Bam*HI) |  |
| pBI121-PsPI-F | ataagggactgaccacccgggTCAATAAAATTGAGTCCAACAATAACAA  (*Sma*I) |  |
